# Dynamic imbalance between cancer cell subpopulations induced by Transforming Growth Factor Beta (TGF-β) is associated with a DNA methylome switch

**DOI:** 10.1186/1471-2164-15-435

**Published:** 2014-06-05

**Authors:** Marion Martin, Pierre-Benoit Ancey, Marie-Pierre Cros, Geoffroy Durand, Florence Le Calvez-Kelm, Hector Hernandez-Vargas, Zdenko Herceg

**Affiliations:** Epigenetics Group, International Agency for Research on Cancer (IARC), 150 rue Albert-Thomas, 69008 Lyon, France; Genetic Cancer Susceptibility Group, International Agency for Research on Cancer (IARC), 150 rue Albert-Thomas, 69008 Lyon, France

**Keywords:** HCC, Tumor-initiating cells, CD133, DNA methylation, TGF-β pathway

## Abstract

**Background:**

Distinct subpopulations of neoplastic cells within tumors, including hepatocellular carcinoma (HCC), display pronounced ability to initiate new tumors and induce metastasis. Recent evidence suggests that signals from transforming growth factor beta (TGF-β) may increase the survival of these so called tumor initiating cells leading to poor HCC prognosis. However, how TGF-β establishes and modifies the key features of these cell subpopulations is not fully understood.

**Results:**

In the present report we describe the differential DNA methylome of CD133-negative and CD133-expressing liver cancer cells. Next, we show that TGF-β is able to increase the proportion of CD133+ cells in liver cancer cell lines in a way that is stable and persistent across cell division. This process is associated with stable genome-wide changes in DNA methylation that persist through cell division. Differential methylation in response to TGF-β is under-represented at promoter CpG islands and enriched at gene bodies, including a locus in the body of the de novo DNA methyl-transferase *DNMT3B* gene. Moreover, phenotypic changes induced by TGF-β, including the induction of CD133, are impaired by siRNA silencing of de novo DNA methyl-transferases.

**Conclusions:**

Our study reveals a self-perpetuating crosstalk between TGF-β signaling and the DNA methylation machinery, which can be relevant in the establishment of cellular phenotypes. This is the first indication of the ability of TGF-β to induce genome-wide changes in DNA methylation, resulting in a stable change in the proportion of liver cancer cell subpopulations.

**Electronic supplementary material:**

The online version of this article (doi:10.1186/1471-2164-15-435) contains supplementary material, which is available to authorized users.

## Background

Hepatocellular carcinoma (HCC) is the major form of primary liver cancer [1], and typically originates in a background of chronic inflammation caused by various factors, such as alcohol consumption, or viral infection (hepatitis B and hepatitis C) [2]. Inflammation is an essential part of the wound-healing response to those risk factors. However, chronic inflammation favors the accumulation of mutations and epigenetic aberrations in hepatocytes, thereby promoting malignant transformation [3, 4]. This process is mediated by chemokines, cytokines, and growth factors secreted by the stromal components of the liver microenvironment [4]. Among those secreted factors, the transforming growth factor beta (TGF-β) has been shown to have a key role that is cell-type dependent and variable during the hepatocarcinogenesis process [5]. In established HCC, TGF-β overexpression is associated with poor prognosis [6–8]. However, characterization of the tumor cells targeted by TGF-β in HCC is still lacking.

As has been shown for other human malignancies, a subpopulation of cancer cells in HCC is known to display a higher tumorigenic potential [9–11]. These so called tumor-initiating cells (TICs), are defined by their self-renewal and differentiation capacity, and have been isolated based on their expression of several cell markers (EpCAM, CD133, CD90, CD44, CD24, CD13, and OV6) [9]. Of these, the surface marker CD133/Prominin1[*PROM1*] has been one of the most consistently reported. CD133 is a transmembrane protein whose function is only partially known [12, 13], but that may represent a marker of a distinct cell subpopulation with defined characteristics. The functional characterization of these cells will increase our understanding of the mechanisms involved in promoting and sustaining liver cancer progression.

Several recent reports suggest a link between TGF-β signaling and liver TICs. Firstly, signaling pathways identified in liver cancer, including TGF-β, are active in isolated liver TICs [14]. Secondly, TGF-β-induced epithelial-mesenchymal transition generates self-renewing cells, a process also implicated in a higher risk of tumor metastasis, as invasiveness and self-renewal are frequently shared features of stem cells, TICs and metastatic cells [15, 16]. Finally, a recent study showed that TGF-β is able to induce the expression of CD133 in liver cancer cell lines together with an increased tumor initiating ability in mice [17]. Together, these studies point towards a specific role for TGF-β in inducing a TIC program in HCC.

DNA methylation is able to stably modify the cell phenotype through cellular division [18]. Because of the relative stability of DNA methylation marks, DNA methylation is a strong candidate mechanism to translate the presence of TGF-β in the cellular microenvironment into persistent changes in phenotype. However, there is still limited evidence of a link between exposure to components of the tumor microenvironment and the induction of stable changes in DNA methylation in target cells.

In this study, we first defined the DNA methylome profile of CD133-expressing liver cancer cells. We then tested the potential association between DNA methylation and the induction of liver CD133+ cells by TGF-β. We show that TGF-β function in this context is intimately linked to a change in DNA methylation profiles, and that this may represent a key process in the establishment of chronic exposure imprints in liver cancer cells.

## Results

### CD133- and CD133+ liver cancer cells differentially express DNA methylation genes

**Figure 1:**
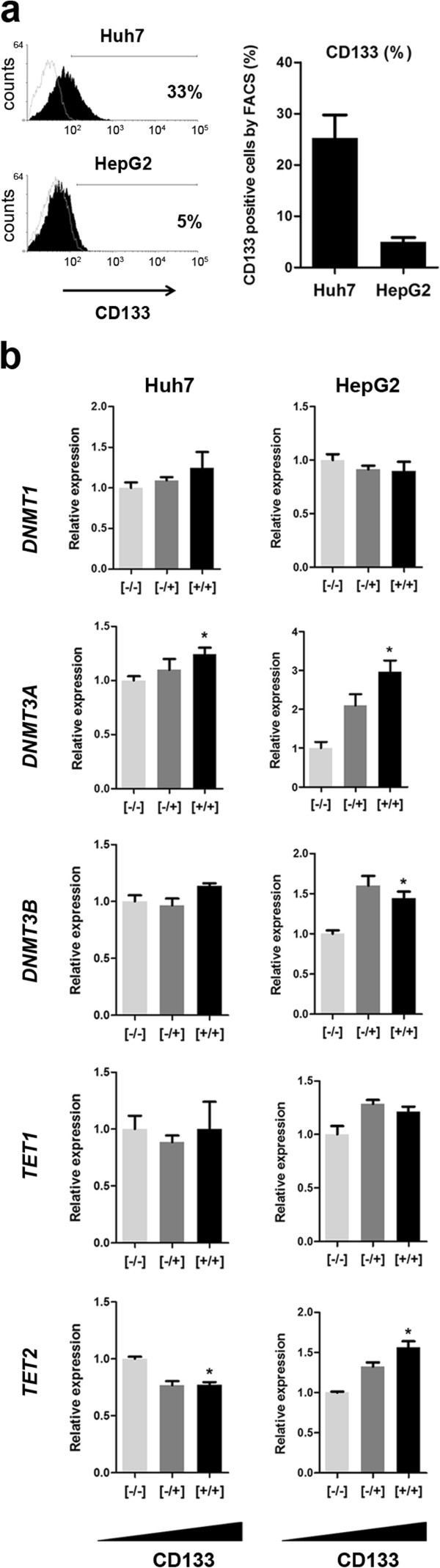
**CD133- and CD133+ liver cancer cells differentially express genes involved in DNA methylation establishment and maintenance. a**. Liver cancer cell lines (Huh7, and HepG2) were assessed for surface expression of CD133 by flow cytometry. The left panel shows a representative histogram for each of the cell lines (black histogram), with background (secondary antibody) represented by the empty histogram (logarithmic scale). The average expression +/- SD from three assays is shown in the right panel. **b**. The same cell lines were sorted using MACS (as described in Methods) and RNA was extracted to measure the expression levels of the genes involved in DNA methylation or demethylation. Expression was normalized to the housekeeping gene *GAPDH*. (*) indicates P value < 0.05.

CD133 is an established marker of TICs in different types of human malignancies, including HCC [12]. To test the notion that this marker distinguishes a cell subpopulation with a distinct DNA methylation program, we characterized two non-related liver cancer cell lines. In a first step, we estimated the frequency of CD133 expressing cells in Huh7 and HepG2 liver cancer cells using fluorescence-activated cell sorting (FACS) against all common CD133 isoforms [12]. The expression of CD133 was evident in both cell lines, with a mean of 5% (SD = 2%) in HepG2, and 25% (SD = 13%) in Huh7 (Figure 1a). Expression of the surface protein positively correlated with CD133 expression at the mRNA level (data not shown). This low to moderate percentage of cells expressing CD133 contrasts with the extreme values of expression that we observed for other molecules such as CD90, CD44 or EpCAM (Additional file 1: Figure S1a). Importantly, expression of CD133 by FACS has been tested at different time points throughout several years, in multiple conditions of cell passages and confluence.The relatively low variability on CD133 expression across time and conditions suggests a dynamic balance between CD133-expressing and non-expressing fractions in both cell lines.

To serve as a basis for exploring a potentially different methylation program in CD133+ liver cancer cells, we studied the expression of genes coding for relevant players of the DNA methylation machinery. This included genes involved in maintenance of DNA methylation (*DNMT1*), de novo DNA methylation (*DNMT3A* and *DNMT3B*) and DNA demethylation (*TET1* and *TET2*). Notably, *DNMT3A* was consistently and significantly overexpressed in both Huh7 and HepG2 cells progressively enriched for CD133 (Figure 1b). In addition, *DNMT3B* was overexpressed in HepG2 CD133-enriched cells, while *TET2* displayed opposite differential expression in CD133-enriched Huh7 and HepG2 cells (Figure 1b). As noted above, the stable balance between the two cell fractions suggests no substantial difference in cell cycle rate between them. Therefore, significant differences in expression, although modest in magnitude, are compatible with true functional differences between the two subpopulations.

Together, these data suggests that CD133 positive and negative fractions grow in a constant proportion within liver cancer cell lines. They differentially express de novo DNA methylation genes (*DNMT3A* in both cell lines, and *DNMT3B* in HepG2) and a subset of genes involved in stemness (Additional file 1: Figure S1b). Functionally, expression of this marker has been associated with an increased tumor-initiating ability and ability to grow in non-attachment conditions, a well known surrogate measure of TIC-like activity. We found that MACS-sorted CD133+ Huh7cells were able to form spheres under non-attachment conditions, in contrast to their CD133- counterpart (Additional file 1: Figure S1c). This was not the case with HepG2 cells, where no sphere formation was observed, possibly due to the lower enrichment of CD133+ cells that was attained using MACS.

### A differential DNA methylome distinguishes CD133- and CD133+ liver cancer cells

**Figure 2:**
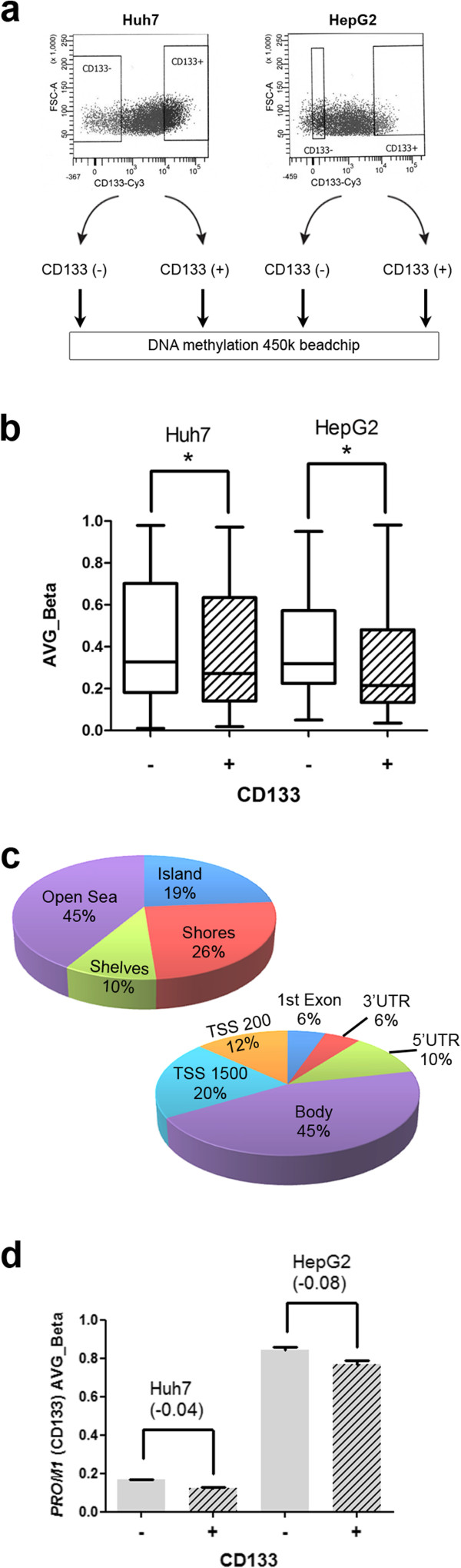
**Differential DNA methylome between CD133- and CD133+ liver cancer cells. a**. Huh7 and HepG2 cells were FACS sorted using CD133 antibody. Gates used to select negative and positive fractions are depicted in the upper panels. Duplicates of each fraction were used for HM450 bead array DNA methylation analyses. **b**. Median methylation (and distribution) for all differentially methylated loci (P < 0.001) distinguishing CD133- versus CD133+ in both cell lines. **c**. Significant loci were distributed according to CpG island relationship as Island, north shore, south shore, north shelf, south shelf, and “Open sea”, and are represented in the upper pie chart. The lower pie chart represents the distribution of significant loci in relation to annotated genes (within 200 or 1500 bp from the TSS, first exon, 3′ or 5′ UTRs, and gene body). **d**. AVG_Beta values obtained from the bead array assay were plotted for one significant CpG site within the *CD133(PROM1)* promoter. The difference in methylation between CD133- and CD133+ cells (delta_Beta) is indicated for each cell line.

The above results support the hypothesis of a phenotypic and functional distinction between CD133+ and CD133- cell fractions. CD133+ cells display a higher expression of de novo DNMTs, and this may be reflected in a differential configuration of their DNA methylome. To study this possibility, we performed a genome-wide DNA methylome analysis in FACS-sorted CD133- and CD133+ fractions from Huh7 and HepG2 cells (Figure 2a). DNA isolated from these fractions was interrogated with the Illumina Infinium HM450 bead array, which covers different genomic features of interest in addition to most human bona fide CpG islands [19]. We first performed unsupervised analyses and found that parental cell line was the main factor defining DNA methylation variation. Therefore, our main analysis compared CD133- to CD133+ fractions accounting for cell of origin (Methods). The class comparison analysis resulted in 823 differentially methylated probes [corresponding to 472 annotated genes] at significant p value (p < 0.001), although relatively high FDRs (FDR = 0.58), probably due to sample and cell line variations. Therefore, for downstream data mining, we increased the stringency of the analyses by further filtering the significant list to keep only those CpG sites where the average differential methylation was at least 5% between the two groups in both cell lines. The resulting 608 differentially methylated probes correspond to 394 RefSeq genes, and represent those CpG sites significantly hypo or hypermethylated in CD133+ cells in both cell lines, relative to their negative counterpart (Additional file 2: Table S1). Most of these probes (n = 510, 84%) were hypomethylated in CD133+ cells, while 98 (16%) were hypermethylated (Figure 2b). An important proportion of differentially methylated loci (45%) were not related to CpG islands (CGI) or their neighboring shelves and shores (“open sea” probes in Figure 2c). For those probes matching annotated genes, we found a significant overrepresentation of differentially methylated loci in the body of the genes (45%). This distribution relative to gene position and CpG island status was similar for hypomethylated sites, while hypermethylated sites were even more enriched in both, open sea (64%) and gene body (57%) probes (data not shown). Supporting the quality of the dataset was the finding of one CpG site within the CD133 (Prominin1 [*PROM1]*) locus among this list of differentially methylated sites. This CpG site was hypomethylated in CD133+ subpopulations from both cell lines, by 4.4% and 8% in Huh7 and HepG2 cells, respectively (Figure 2d).

After having identified differentially methylated CpGs and the genes associated with these sites, we next aimed to identify the pathways that are specifically altered in CD133+ cells. To this end, we performed pathway analysis considering methylome profiles of both cell lines together or independently. Notably, in both cases there was enrichment in pathways previously associated with tumor initiating cell activity, such as Jak-STAT, Notch, Wnt and Akt (Additional file 3: Table S2). Other pathways included actin cytoskeleton, focal adhesion, and cell adhesion. In addition, there was a significant overrepresentation of inflammatory pathways, such as NFkB, p38, TNF, and TGF-β signaling pathways.

In summary, our data shows that CD133+ and CD133- liver cancer cells display a different DNA methylome. In spite of the cell line specific profiles, the data suggests a common CD133+ methylome signature, which includes the *PROM1* gene itself. In addition, the methylome of CD133+ cells is characterized by a global reduction in DNA methylation relative to their CD133- counterpart, with an overrepresentation of non-CGI CpG sites. For those differentially methylated sites related to annotated genes (and mainly found in the gene bodies), there was an association with TIC- and inflammation-related pathways. These findings suggest that specific DNA methylation profiles are associated to the phenotype and functionality of these cell subpopulations.

### TGF-β, but not IL-6, induces CD133 expression in a stable fashion

**Figure 3:**
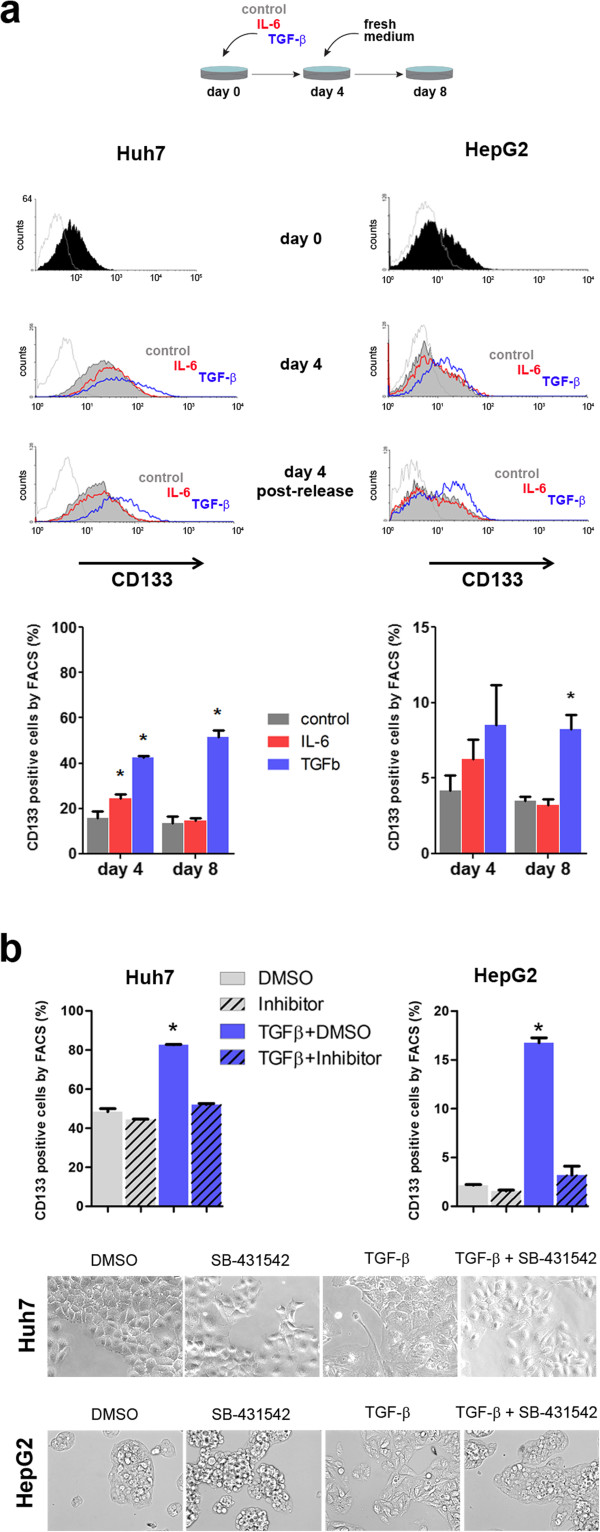
**TGF-β, but not IL-6, induces CD133 expression in a stable fashion. a**. Experimental design is indicated in the upper panel. Huh7 and HepG2 cells were grown in control culture conditions (depicted in gray text and lines), or exposed to 10 ng/ml IL-6 (red) or 10 ng/ml TGF-β (blue) for 4 days. Cells plated in parallel, had their medium replaced by control culture medium and were left in culture for additional 4 days. FACS expression of surface CD133 protein is shown for day 0, day 4, and day 8 (4 days treatment + 4 days post-release) for all conditions, in logarithmic scale. Histograms are shown for one representative replicate in the middle panel. Mean +/- SD is shown for three biological replicates in the lower panel barplots. **b**. TGF-β type I receptor antagonist SB 431542 was used at 2uM, alone or in combination with 10 ng/ml of TGF-β, and DMSO used as control. CD133 expression was studied by FACS after 4 consecutive days of exposure to each experimental condition. (*) indicates P value < 0.05 relative to all other conditions, for both cell lines. Representative phase contrast images are shown in the lower panels.

It has been reported that TGF-β exposure increases the percentage of CD133+ cells in the Huh7 cell line [17], although the underlying mechanism remains largely unknown. We thus aimed to investigate whether this observation is consistent in two independent cell lines and compatible with an epigenetic mechanism (i.e. persistent through cell division). Importantly, both Huh7 and HepG2 cells, express the receptor for TGF-β (TGFBRII) at similar levels, and respond to TGF-β by phosphorylating the receptor-dependent SMAD3 (Additional file 4: Figure S2a and S2b). In addition to TGF-β, we performed a set of parallel experiments with the pro-inflammatory cytokine interleukin 6 (IL-6), which has also been associated with HCC risk [20]. To this end, we selected commonly used cytokine concentrations that induced morphological changes after 4 days of treatment in both cell lines (in the case of TGF-β), but did not have any effect on cell viability (Additional file 4: Figure S2c and S2d). As expected, TGF-β exposure during 4 days induced a two-fold increase in the percentage of CD133+ cells in Huh7 and HepG2 cells (Figure 3a). Interestingly, IL-6 treatment also induced an increase in CD133 positivity in both cell lines, although the increase was comparatively mild (approximately 50% increase) (Figure 3a). Next, we analyzed the persistence of the effect in CD133 expression induced by both cytokines. To this end, we treated both cell lines as in the previous experiment. After 4 days, cell culture medium was replaced by standard medium, and cells were left in culture for additional 4 days. Cells were collected and screened for CD133 expression using FACS. Notably, only cells treated with TGF-β showed a persistent increase in the percentage of CD133+ cells, of similar magnitude to the increase observed at day 4 (Figure 3a). Importantly, only TGF-β exposure was able to induce a significant increase in the expression of CD133 at the transcriptional level in both cell lines (8 and 6 fold increase for Huh7 and HepG2, respectively) (Additional file 5: Figure S3b).

TGF-β is a member of a large family of pleiotropic cytokines that signal through a receptor complex comprising a diversity of type I and type II serine/threonine kinases. The recombinant TGF-β1 used in our assays is expected to bind the activin receptor-like kinase (ALK)5 (the TGF-beta type I receptor) [21]. To rule out unspecific effects of this treatment, we used the small molecule inhibitor SB-431542, which targets ALK5 and ALK5-related type I receptors, with no effect on other family members that, for example, recognize bone morphogenetic proteins (BMPs) [22]. By using this specific inhibitor of TGF-β pathway, we were able to abrogate the effect of TGF-β in inducing CD133 expression (as well as the morphological changes) in both cell lines (Figure 3b and Additional file 4: Figure S2e). Therefore, the ability to induce CD133+ cells is specific and fully dependent on TGF-β type I receptor signaling in both, Huh7 and HepG2 cells (Figure 3b).

Together, these findings suggest that TGF-β is able to specifically and stably induce CD133 expression (in contrast to the milder and transient effect of IL-6), an observation consistent with epigenetically-induced phenotype persistence.

### De novo induction of CD133 by TGF-β is associated to an increased expression of DNMT3 genes

**Figure 4:**
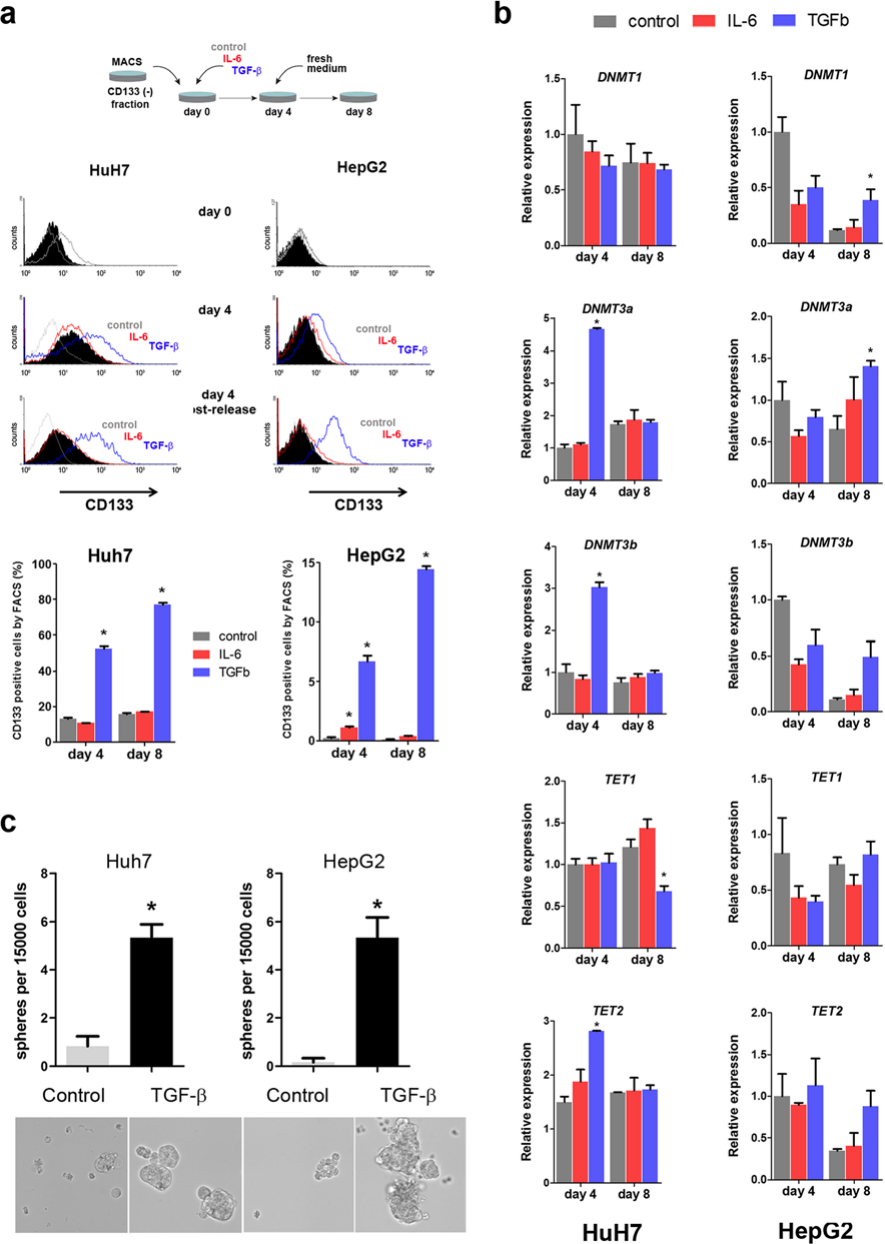
**de novo CD133 induction by TGF-β correlates with overexpression of DNMT3 genes. a**. the experiment in Figure 3A was repeated after MACS-sorting to enrich in CD133 negative cells, as depicted in the upper panel. Levels of CD133 expression were close to 0%, as shown in the upper histograms for both, Huh7 and HepG2 cells. Mean from three replicates is shown in the lower panels. (*) indicates P value < 0.05 relative to control conditions. **b**. sphere formation assays were performed in non-attachment plates, after exposure to TGF-β during 4 days. Spheres were counted after 6 days of growth in hepatosphere medium w/o TGF-β. **c**. Huh7 (left panels) and HepG2 (right panel) cells were treated as in (**a**), RNA was extracted and qRT-PCR was performed for genes involved in DNA methylation or demethylation. Expression was normalized to housekeeping gene *GAPDH*. (*) indicates P value < 0.05 relative to non-treated cells at the corresponding time point.

The increase in CD133 positivity induced by TGF-β can be explained by a switch in the expression of CD133, or an increased rate of growth specifically in the smaller CD133+ fraction of cells. To distinguish between these two possibilities, we repeated the previous experiment in cells negative for CD133 expression, selected by depletion of CD133+ cells using MACS (Methods). In both cell lines, TGF-β was able to significantly induce a population of CD133+ cells, evident after 4 days of treatment (Figure 4a). Also in this case, we replaced the medium after 4 days, and let the cells grow in the absence of cytokines for additional 4 days. After these additional 4 days, the increase in CD133 positive fraction was higher, relative to the one observed at day 4, for both cell lines (Figure 4a, and Figure 3a for comparison). Importantly, although there was a spontaneous induction of a CD133+ fraction in Huh7 cells (from 0 to 20% after 4 days), this percentage did not significantly change at day 8, and is similar to what is found in untreated Huh7 cells in basal conditions. As discussed above, this suggests a dynamic balance between the CD133 negative and positive fractions in this cell line. The surface expression of CD133 remained close to zero in HepG2 control cells. This finding suggests that TGF-β is able to induce the expression of CD133 surface protein, and not an increased proliferation of CD133+ cells. This is also supported by the expected lower rate of proliferation of cells treated with TGF-β (Additional file 5: Figure S3a). Similar to our previous experiment, under these conditions IL6 only showed a transient effect (Figure 4a).

After having shown that TGF-β may be able to induce a de novo fraction of CD133+ cells, we asked whether this effect correlated with a differential expression of DNA methylation players, as we have shown that CD133+ cells overexpress *DNMT3* genes in basal culture conditions (Figure 1b). DNMTs and *TET2* displayed a significant increase in mRNA expression in at least one of the two cell lines, while *TET1* was underexpressed after 4 days of release from TGF-β exposure (Figure 4b). As shown for the basal CD133-expressing cells (i.e. those isolated from untreated HCC cell lines), the most consistent finding was the statistically significant overexpression of *DNMT3A* in both cell lines after TGF-β treatment. Of note, in none of the conditions of study was IL-6 exposure able to induce statistically significant changes at the mRNA expression level of genes related to DNA methylation/demethylation (Figure 4b).Combined, these data shows the ability of TGF-β (in contrast to IL-6) to induce a stable de novo fraction of CD133-expressing cells in two independent liver cancer cell lines. This induction correlates with a functional characteristic of basal CD133+ cells, which is the increased ability to grow under non-attachment cell culture conditions (Figure 4c). Moreover, the persistence of CD133 induction by TGF-β (4 days after release from TGF-β treatment, and at least two cell divisions apart) suggests an epigenetic process is taking place, in contrast to the transient induction of CD133 by IL-6.

### Cell subpopulation switch induced by TGF-β correlates with a differential DNA methylome

**Figure 5:**
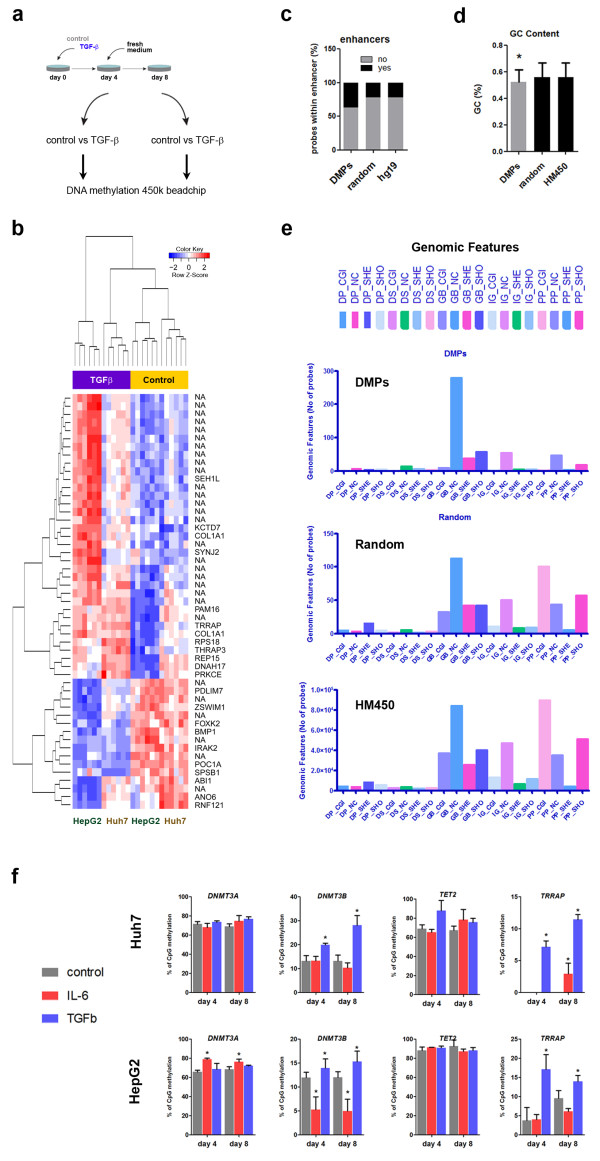
**Cell subpopulation imbalance induced by TGFβ correlates with a methylome reconfiguration. a**. Huh7 and HepG2 cells were treated with TGF-β for 4 days, or 4 + 4 days post-release, as described above. Biological triplicates were used to assess DNA methylation changes with HM450 arrays. **b**. heatmap represents probes differentially methylated (FDR < 0.05) with a delta-beta of at least 20% (n = 41) between control and TGF-β treated cells, in both cell lines, and both time points. For a full list of DMPs (FDR < 0.05 and delta-beta of at least 10%) see Table S3. Blue indicates lower methylation, and red indicates higher methylation. The unsupervised clustering distinguishes TGF-β from control conditions regardless of cell line or time of exposure. **c**. 555 DMPs (FDR < 0.05, delta > 10%) were mapped to known enhancer regions in the human hg19 genome assembly. Enrichment is observed in DMPs, relative to a random selection of the same number of probes and the total of HM450 probes mapping to an enhancer (hg19). **d**. in similar way, GC content was compared between DMPs, random probes, and all HM450 probes (HM450). (*) indicates a significantly lower GC content in DMPs relative to any of the other probe lists. **e**. DMPs were distributed according to their relationship to CpG islands (CGI: islands, SHO: shores, SHE: shelves, or NC: non-CpG islands) or genes (DP: distal promoter, DS: distal sequence, GB: gene body, IG: intergenic, and PP: proximal promoter), as described in Materials and Methods. **f**. A selection of significant loci were validated by pyrosequencing (as described in Materials and Methods), in both cell lines. (*) indicates P value < 0.05 relative to non-treated cells at the corresponding time point and cell line. IL-6 was included for comparison.

Having shown that CD133+ cells display a unique DNA methylome, and that TGF-β is able to induce a de novo CD133+ fraction of cells, we decided to study the DNA methylome induced by TGF-β exposure. To this end, we used HM450 bead arrays to interrogate DNA methylation changes induced by 4 days of TGF-β exposure in both, Huh7 and HepG2 cells (Figure 5a). In addition, to define the epigenetic persistence of TGF-β effects, we included the DNA from cells released 4 days into normal cell culture medium after the TGF-β treatment. As described above for the DNA methylation profile of CD133-expressing cells, the methylome of Huh7 and HepG2 cells are clearly distinguishable, independently of the experimental conditions. However, in addition to cell type-specific changes we were able to observe genome-wide changes induced by TGF-β in a cell type-independent fashion. To define a TGF-β-induced DNA methylation signature, we focused on those loci that were significantly hypo or hypermethylated in both cell lines. In addition, we were interested in those changes that were persistent through cell division and stable in the absence of TGF-β. Therefore, we selected significant loci (FDR < 0.05) that were differentially methylated at both, 4 days of treatment and 4 additional days after release. Finally, we selected those CpG sites that reached an average difference of at least 10% between control and TGF-β conditions (Figure 5b). 555 differentially methylated positions (DMPs) fulfill all criteria, with 115 hypomethylated after TGF-β exposure (21%) and a great majority hypermethylated (n = 440, 82%) (Additional file 6: Table S3), including multiple sites on intergenic regions. Hypermethylated DMPs included loci in *TRRAP, COL1A1, DNAH17, ARID1B, ONECUT2*, and *DNMT3B*. Hypomethylated DMPs included loci in *TGFB2, BMP1, IRAK2*, and *FOXK2*. Interestingly, there was an enrichment of DMPs on enhancer regions (Figure 5c). In addition, we found a significantly lower GC content in DMPs when compared to a random selection of probes or to the overall GC content of HM450 probes (Figure 5d). To study the genomic context in more detail we analyzed the overlap of DMPs with genomic features, as previously described [23]. These annotations consider the relationship with CpG islands (CGI) (i.e. islands, shores, shelves, or open sea) and the location with respect to the gene (i.e. distal and proximal promoter, gene body, distal, and intergenic sequences). An important proportion of probes in HM450 bead arrays target proximal promoter CGIs and shores, and non-CGI gene bodies (HM450 in Figure 5e). A similar distribution is observed when overlapping a randomly generated list of sites (of the same size of the DMP list, n = 555) with the genomic features. However, there is a striking under-representation of promoter CGIs and shores in the DMP list (Figure 5e). Instead, a great majority of DMPs are found within the gene bodies in non-CGI regions, in agreement with our previous observation of low GC content (Figure 5d).

Next, we searched for differentially methylated regions (DMRs) in response to TGF-β, and common to both cell lines. We used as criteria for DMR the presence of at least two neighboring DMPs within a minimum gap of 100bp at a significant FDR < 0.05. Using these criteria we obtained 18 DMRs, oscillating from 2 to 6 DMPs on each (Table 1). Interestingly, one of these DMRs is comprised of 3 CpG sites corresponding to the body of the *DNMT3B* gene. Indeed, the majority of DMRs (16 out of 18) were found outside of gene promoters. DMPs in *DNMT3B* and *TRRAP* were validated using an independent quantitative method, bisulfite DNA pyrosequencing (Figure 5f). Both assays confirmed hypermethylation in response to 4 days of TGF-β and 4 days after release, in the two cell lines. In contrast, no differential methylation was observed in pyrosequencing assays performed on *DNMT3A* and *TET2* in response to TGF-β, although increased methylation at the *DNMT3A* locus was observed after IL-6 exposure in HepG2 cells (Figure 5f).

**Table 1:** **Differentially methylated regions (DMRs) after TGF-β exposure in two liver cancer cell lines and two time points (4 days treatment and 4 additional days post-release)**

CHR	Start	End	# probes	EntrezID	Symbol	Distance2TSS	Promoter
chr1	2.26E + 08	2.26E + 08	3	2052	EPHX1	0	TRUE
chr10	1.35E + 08	1.35E + 08	6	10844	TUBGCP2	2052	FALSE
chr12	1.21E + 08	1.21E + 08	4	84747	UNC119B	6288	FALSE
chr13	92002338	92002454	2	407975	MIR17HG	2264	FALSE
chr15	78286548	78286614	2	91450	LOC91450	0	TRUE
chr16	87437787	87437924	2	81631	MAP1LC3B	11986	FALSE
chr17	48270042	48270097	3	1277	COL1A1	8903	FALSE
chr17	48275324	48275919	2	1277	COL1A1	3081	FALSE
chr17	73631586	73631785	2	643008	SMIM5	2072	FALSE
chr17	80560479	80560634	3	3607	FOXK2	6821	FALSE
chr19	23278023	23278036	2	1E + 08	ZNF730	-21741	FALSE
chr19	24232204	24232430	2	9534	ZNF254	15957	FALSE
chr20	31366437	31366486	3	1789	DNMT3B	16246	FALSE
chr5	52096641	52096811	2	53918	PELO	12867	FALSE
chr6	33241410	33241770	4	6222	RPS18	1558	FALSE
chr7	1.35E + 08	1.35E + 08	3	800	CALD1	111142	FALSE
chr7	1.57E + 08	1.57E + 08	3	10049	DNAJB6	1459	FALSE
chr9	1.33E + 08	1.33E + 08	2	23413	NCS1	34026	FALSE

See Methods for definition of DMR.

To gain a better insight on the consequences of TGF-β-induced methylome switch on the phenotype, we performed a whole genome expression analysis in both, Huh7 and HepG2 cells. We chose the 8-days time point (4 days of TGF-β treatment + 4 days post-release), considered in our model as the one defining long-term, stable changes induced by this cytokine. Expression analysis showed an expected profile of gene expression in both cell lines, including known TGF-β targets (Additional file 7: Table S4, Additional file 8: Figure S4c for qRT-PCR validations). In addition, “type I transforming growth factor beta receptor binding” was the first gene ontology category at the molecular function level (Additional file 7: Table S4). However, when intersecting the expression (two-fold change with an FDR < 0.05) and methylation (555 DMPs) significant gene lists, there was no significant overlap. As the effect of a specific methylation change on gene transcription is known to depend on the genomic location [24], we plotted all expression and methylation data, and analyzed separately CpG island and non-CpG island sites. As expected, no obvious correlation can be seen when plotting simultaneously all genes, independently of genomic location. However, hypermethylation within the gene bodies positively correlated with gene expression (Additional file 8: Figure S4a and S4b). In addition, we observed a small but significant overlap of 30 genes when intersecting the CD133 and the TGF-β methylation signatures (p = 0.0013).

Our data shows that the effect of TGF-β in liver cancer cell lines comes along with a remarkable switch of the DNA methylome at multiple loci. This reconfiguration is stable and common to two independent cell lines, and affects a significant proportion of enhancer regions and GC poor regions on gene bodies, which in some cases correlates positively with gene expression. The TGF-β “methyl-sensitive” signature described here includes DNA methylation players themselves and a number of TGF-β pathway loci, indicating a potential role for DNA methylation in establishing a TGF-β-induced phenotype switch in these cells. These results suggest that basal CD133+ cells and TGF-β-induced CD133+ cells only share a limited subset of their methylome. An important fraction of TGF-β methyl-sensitive CpG sites are not differentially methylated in CD133+ cells.

### Kinetics of DNA methylation changes at TGF-β methyl-sensitive sites

**Figure 6:**
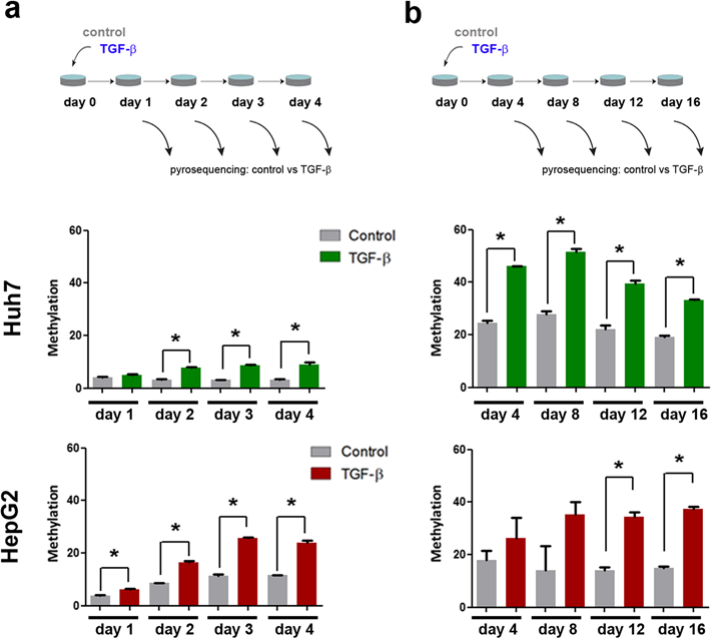
**Kinetics of DNA methylation after TGF-β treatment. a**. Huh7 and HepG2 cells were exposed to TGF-β for shorter time points (1 to 4 days), followed by DNA extraction, bisulfite modification, and pyrosequencing analysis of one CpG site in the body of *TRRAP* gene (a DMP showing hypermethylation in response to TGF-β, as seen in Figure 3b). **b**. in a similar way, both cell lines were studied at longer time points after release from 4 days of TGF-β treatment. Cells were followed during 12 days post-release and displayed a rate of proliferation close to non-treated cells. (*) indicates P value < 0.05 relative to non-treated cells at the corresponding time point and cell line.

Our methylome analyses have defined a subset of DNA sites differentially methylated in response to TGF-β exposure in a stable and cell line-independent fashion. We performed two independent experiments to monitor the dynamics of these DNA methylation changes induced by TGF-β. First, we studied how early after TGF-β stimulation we were able to observe methylation differences. To this end, we extracted DNA every 24h during 4 consecutive days in TGF-β treated and control cells grown in parallel. Specifically, we studied the methylation of one CpG site in the gene body of *TRRAP*, selected from our list of significant hits as a representative of the pattern most consistently observed in our dataset: differential methylation of single sites (non-CpG island sites) in the body of the genes. Basal methylation of *TRRAP* was relatively stable within this experiment (Figure 6a). In contrast, *TRRAP* methylation is significantly increased as early as 1 day in HepG2 cells and 2 days in Huh7 cells after TGF-β exposure. This difference remains constant up to the 4th day of TGF-β treatment in both cell lines.

In the second experiment we aimed to assess the stability of the methylation changes at longer time points. To this end, cells were treated with TGF-β during 4 consecutive days (Figure 6b). After this time cell culture medium was replaced by standard medium, and control and treated cells were followed for DNA extraction every 4 days until day 16 (corresponding to day 12 post-release from TGF-β exposure). First, we observed differences on the basal methylation of *TRRAP* when comparing to the previous experiments, especially in Huh7 cells (Figure 6a). This could be the result of differences on the efficiency of bisulfite conversion between experiments, but also differences between cell lines from various origins, as the Huh7 cells used for the second experiment were obtained from an independent laboratory. Regardless of basal difference, we observed that the increased methylation of *TRRAP* was replicated in both cell lines. Furthermore, significantly higher methylation levels persisted up to 12 days post-release from TGF-β, with no apparent change in its magnitude. Importantly, cell growth was not significantly affected after TGF-β release, and we estimate that at least 8 cell duplications took place during this time.

In summary, DNA methylation changes in a typical TGF-β methyl-sensitive locus in the *TRRAP* gene body can be observed as early as 1-2 days following TGF-β exposure, and persist through cell division for at least 16 days without apparent change in magnitude.

### Silencing of de novo DNA methyl-transferases impair the effect of TGF-β at methyl-sensitive sites

**Figure 7:**
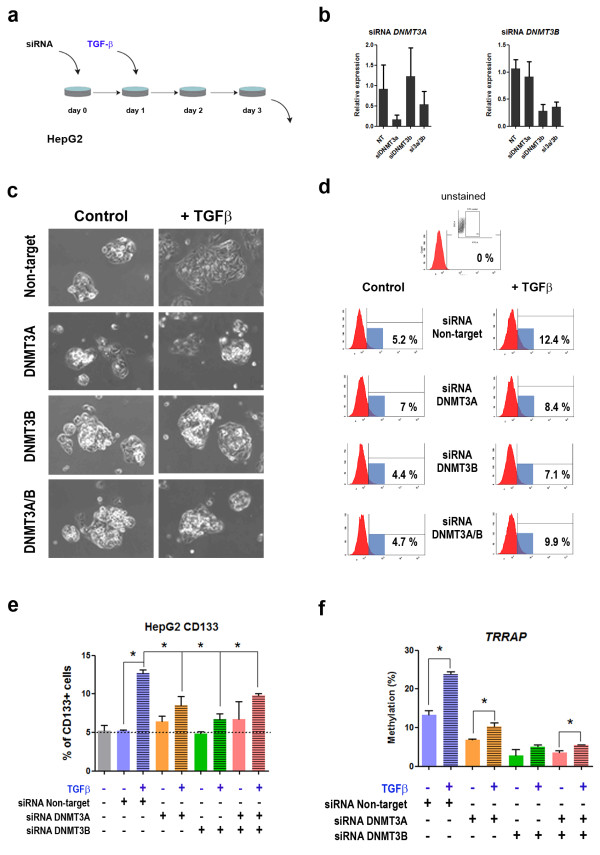
**Silencing of DNMT3s impairs the effects of TGF-β on liver cancer cells. a**. HepG2 cells were transfected with 20nM of a non-targeting siRNA, or a pool of siRNAs against *DNMT3A*, *DNMT3B*, or a combination of both. One day after transfection cell medium was replaced by standard culture medium or medium containing 10nM TGF-β for each condition. Two days after treatment with TGF-β cells were collected from all conditions for morphological evaluation, CD133 FACS expression analysis, RNA and DNA extraction. All transfections were performed in triplicate wells. **b**. qRT-PCR was performed to assess the efficiency and specificity of the transfections. Higher efficiency was obtained with siRNA against DNMT3B alone or in combination with DNMT3A. **c**. Representative Phase contrast images for all conditions (compare with Figure S2c for the morphology of non-transfected HepG2 cells in basal conditions and in response to TGF-β). **d**. cells were collected after treatment and processed immediately for analysis of CD133 surface expression using FACS. Secondary antibody staining (with no primary CD133 staining) is shown as control in the top panel, and was used as a reference for the gates shown below. The plots show one representative histogram for each condition, and the corresponding percentage of CD133+ cells (compare with Figure 1a and Figure 7e for the basal expression and variation in HepG2 non-transfected cells). **e**. Mean + SEM for CD133 FACS expression in triplicates of each condition. A non-transfected control was also included in this analysis. Asterisks depict the significance between control and TGF-β -treated cells transfected with non-targeting siRNA, and all other TGF-β -treated conditions compared to non-targeting TGF-β -treated cells. **f**. DNA was extracted and bisulfite modified for pyrosequencing of TRRAP. (*) indicates P value < 0.05 relative to non-treated cells within each experimental condition.

To have an insight into the causal role of DNA methylation on the cancer cell population switch induced by TGF-β, we performed an siRNA experiment to silence de novo DNA methyl-transferases in the context of TGF-β exposure in HepG2 cells using a pool of siRNAs (Methods). The main readout of our experiment was the ability of TGF-β to induce a 2- to 3-fold change in the expression of CD133 by FACS analysis of HepG2 cells (as shown in Figures 3a and 4a). Considering that the previous kinetics experiment showed *TRRAP* hypermethylation at 2 days post- TGF-β treatment, we started treatment with TGF-β 24h post-transfection and collected DNA two days later (Figure 7a). siRNA efficiency checked by qRT-PCR (Figure 7b) showed an effect of both siRNAs when used alone or in combination, although siRNA against *DNMT3B* displayed a better efficacy of silencing the corresponding transcript. Transfection with a non-targeting siRNA did not affect the morphology of HepG2 cells, typically growing as refractive, well-delimited colonies (Figure 7c). The expected response to TGF-β was also not affected by the non-targeting siRNA, with the expected loss of the refractive colonies and the presence of flattened enlarged cells. Transfection with DNMT3A or DNMT3B siRNAs, or combination of both did not have any effect on cell morphology. However, in contrast to the non-targeting control, there was no response to TGF-β exposure and most cells remained as refractive colonies in all other conditions without significant loss of cell viability (Figure 7c). Similarly, non-targeting siRNA did not influence the expected increase in CD133+ expression by FACS (from 5.2% to 12.7% in average) (Figure 7d and 7e). There was no effect of siRNA against DNMT3s alone or in combination on the basal expression of CD133. However, the response to TGF-β was significantly impaired in all conditions (Figure 7d and 7e). Therefore, the morphological “rescue” was paralleled by the CD133 phenotype.

Finally, we tested if, under the same conditions, DNA methylation changes in response to TGF-β were also impaired after DNMT3 silencing. *TRRAP* methylation is induced after 2 days of TGF-β treatment in HepG2 cells, an effect not influenced by the transfection of a non-targeting siRNA (Figure 7f). However, all further experimental conditions significantly reduce *TRRAP* methylation in the absence of TGF-β. In addition, the hypermethylation induced by TGF-β exposure was impaired, especially after transfection with siRNA against *DNMT3B*. Together, these results suggest that de novo DNA methyl-transferases are involved in the effect of TGF-β at different levels: morphology, distribution of cell subpopulations, and DNA methylation. The hypomethylation observed in absence of TGF-β also suggests a role for DNMT3A and DNMT3B in maintenance of DNA methylation at the *TRRAP* locus.

## Discussion and conclusions

In the present report, we comprehensively describe the DNA methylome of CD133+ and CD133- liver cancer cells. We used two non-related HCC cell lines to isolate pure populations of CD133- and CD133+ cells for DNA methylome assays. As has been previously reported, CD133+ cells isolated from liver cancer cell lines (including those used in the present study), are functionally distinct cells with increased ability to induce tumors in animal models [14]. These findings are in line with clinical studies reporting poor prognosis for those HCC cases displaying higher proportions of CD133-expressing cells. Although testing the tumor-initiating or metastasis-initiating ability of CD133+ cells was beyond the scope of our study, our results suggest that these cells display a differential DNA methylation signature. Whether DNA methylation is fundamental in establishing the cellular programs defining the main characteristics of these cells should be the focus of future studies.

Recently, the prognostic implications of TGF-β pathway activation in HCC have been linked to the ability of this signaling pathway to induce metastatic behavior in a fraction of HCC cells [25]. An additional link between liver TICs and TGF-β in HCC has been the recent demonstration that TGF-β is able to increase the proportion of CD133+ cells in vitro [17]. Here, we were able to reproduce and extend those previous observations. We showed that TGF-β is able to increase CD133 expression at the protein and mRNA level in two non-related HCC cell lines. The effect induced by TGF-β is stable, as opposed to the transient effect of the proinflammatory cytokine IL-6. We show that this effect depends on specific signaling through TGF-β type I receptor and is independent of increased cell proliferation of CD133+ cells. By using CD133-depleted cellular fractions, we suggest that TGF-β is able to induce de novo expression of CD133. Furthermore, this induction of CD133 cells correlates with an increased ability to grow in non-attachment conditions, a surrogate functional assay for stem/TIC properties.

Both, basal CD133-expressing cells and TGF-β-induced CD133+ cells, expressed increased levels of the de novo DNA methylation transcripts, *DNMT3A* and *DNMT3B*. This led us to further explore the ability of TGF-β to induce DNA methylation changes at the genome-wide level. We were able to show cell line-independent changes in DNA methylation induced by TGF-β in a stable fashion, suggesting an epigenetic mechanism involved in the establishment of a cellular program. The methylome of TGF-β -treated cells only partially overlapped with the methylome of CD133+ cells in basal conditions. This suggests that TGF-β may not only induce CD133 expression (or an increase in the CD133-expressing subpopulation), but also a defined DNA methylation profile. Further studies at longer time points and analyses of isolated CD133 negative and positive cells may shed light on the ability of TGF-β to imprint a DNA methylation signature independently of the induction of CD133 expression.

Notably, although TGF-β is known to induce DNA methylation changes at discrete loci [26–28], little evidence existed to date of a genome-wide level of TGF-β-methyl-sensitive loci. Specifically, several previous reports were focused on chromatin changes associated with epithelial-mesenchymal transition (EMT). EMT is a developmental process that involves actin cytoskeleton reorganization and loss of apical–basal polarity and cell-to-cell contact, and like other developmental processes it involves epigenetic reprogramming [29]. However, both at physiological and pathological levels, EMT has been mainly linked to widespread changes of histone marks or histone modifiers, in addition to the well-known role of defined transcription factors [30–32]. Interestingly, gene-specific changes in DNA methylation have been correlated with the ability to maintain epigenetic silencing of critical EMT genes [27]. In this sense, it has been suggested that DNA methylation is involved in the process of fixing the switch between epithelial and mesenchymal phenotypes. In our conditions, TGF-β induced a loss of E-Cadherin expression in both cell lines, but not an evident increase in N-Cadherin, which is a known marker of EMT (Additional file 4: Figure S2f). However, our results are consistent with a model of persistent changes in DNA methylation induced by TGF-β. Indeed, our experimental design was intended to reproduce an epigenetic process, by selecting only those changes in DNA methylation that survived cell division. Whether this effect of TGF-β is specific of transformed cells will require further studies.

Regardless of considerations on the targeted cells or the effect of the cell cycle or cell subtypes, we describe what seems to be a unique pattern of differential methylation. Our TGF-β signature (stable after TGF-β removal, and common to two liver cancer cell lines) was characterized by a reduced GC content, and enrichment at enhancer regions. Moreover, the under-representation of promoter CGIs and enrichment on gene bodies (together with the positive correlation with gene expression in a subset of these sites), may indicate specific mechanisms of gene regulation through DNA methylation. For example, changes in DNA methylation on these TGF-β methyl-sensitive loci may drive differential enhancer activity or alternative transcriptional events, as has been suggested in other contexts [23]. Whether similar findings occur in response to other cytokines is not currently known.

In summary, our data support and reinforce several previous studies that have pointed to an association between TICs, and TGF-β. In addition, we provide a mechanistic insight into the process that may lead to the stable change in cancer cell subpopulations. Our study demonstrates that a key cytokine involved in HCC progression, TGF-β, is able to epigenetically induce a dynamic imbalance between cell subpopulations. The results reported here are in agreement with a model in which DNA methylation plays a pivotal role in establishing the cellular program of liver cancer cell subpopulations. The dynamics of a related process has recently been shown for CD44+ breast cancer stem cells [33]. However, in our model, the effect of TGF-β is persistent (as compared to the effect of another cytokine, IL-6) and therefore epigenetically acquired. Motivated by these observations, the mechanistic evidence of an active interplay between TGF-β and the DNA methylation machinery should be an exciting focus of future studies.

## Methods

### Cell culture and treatments

Huh7 and HepG2 cells (American Type Culture Conditions) were cultured in DMEM medium (Gibco) at 37°C and 5% CO2, and were regularly tested for mycoplasma contamination (MycoAlert detection kit, Lonza).

For cytokine treatments, cells were plated and allowed to adhere before adding medium containing 10ng/ml final of IL-6 or TGF-β1 (recombinant human, Peprotech). For inhibition experiments, cells were treated with 2 μM SB-431542 (Sigma-Aldrich) alone or in combination with TGF-β1.

For spheres formation assay, hepatosphere medium was prepared as previously reported [34]. Spheres were counted after 5 or 6 days.

siRNA non-targeting and pool siRNAs against DNMT3A and DNMT3B (Dharmacon, On-Target plus siRNA) were transfected at 20nM using RNAiMAX lipofectamine (Life Technologies) as recommended by the manufacturer. Cells were washed and medium was replaced 12 hours after transfection.

### Fluorescence Activated Cell Sorting (FACS)

Cells were labeled with antibodies against CD44, CD133 (AC133), EpCAM, CD90 or TGFBRII (Additional file 9: Table S5). Secondary antibodies were conjugated alternatively with FITC, Cy3 or Alexa750.

To study cell cycle progression, bromodeuxyridine (BrdU) (Sigma) incorporation and DNA content were simultaneously assessed, as previously described [35]. Fluorescent events were captured using FACS instrument (FACSCanto II, BD Biosciences), and analyzed using BD FACSdiva 6.0 (BD Biosciences software) and WinMDI software (version 2.9).

### Magnetic Activated cell sorting (MACS)

Huh7 and HepG2 cells were depleted or enriched for CD133+ cells using magnetic-activated cell sorting (MACS, Miltenyi Biotec), with some adaptations to the manufacturer’s instructions. Cells were incubated 30 min at 4°C with FcR blocking reagent, followed by 15 min incubation with MicroBeads conjugated to monoclonal anti human CD133 antibodies. After washing, cell suspension was applied onto a pre-rinsed LS column placed in the magnetic field of a MACS separator. For CD133+ depletion, flow-through the LS column containing unlabelled cells was collected. For CD133+ enrichment, the column was removed from the separator and placed on a 15 ml collection tube. Labeled cells were collected by firmly pushing the plunger in the column. To increase purity of CD133+ cells, the eluted fraction was enriched a second time over a new LS column. For each experiment, aliquots were kept to test by FACS the efficiency of the enrichment.

### Bisulfite modification and pyrosequencing

To quantify the percentage of methylated cytosine in individual CpG sites, we performed bisulfite pyrosequencing, as previously described [36]. For samples processed for Infinium bead arrays, the conversion was performed on 600 ng of DNA using the EZ DNA methylation Kit (Zymo Research) and modified DNA was eluted in 16 ul of water. Quality of modification was checked by PCR using modified and unmodified primers for *GAPDH* gene. Pyrosequencing assays (primers for PCR, sequencing primers and regions) are described in Additional file 9: Table S6.

### Bead array methylation assays

Methylation profiles of the different samples were analyzed using the HM450 Infinium methylation bead arrays (Illumina, San Diego, USA). Briefly the HM450 beadchip interrogates more than 480,000 methylation sites [19]. The analysis on the bead array was conducted following the recommended protocols for amplification, labeling, hybridization and scanning. Each methylation analysis was performed in duplicate (for CD133+ versus CD133- samples) or in triplicate (for all other methylome analyses).

### Whole genome expression array

Total RNA was isolated using the TRIzol Reagent (Invitrogen) according to the manufacturer’s instructions. RNA quantity and quality were assessed with a ND-8000 spectrophotometer and bioanalyzer. 500 ng of total RNA was used for each Human HT-12 Expression BeadChips (Illumina), as previously described [37]. 10 candidate genes were selected for validation using quantitative RT-PCR. Four different housekeeping genes (*HPRT1, GAPDH, SFRS4* and *TBP1*) were alternatively used for internal control. The different primers used are listed in Additional file 9: Table S7.

### Immunoblotting and immunofluorescence

Protein extraction and immunoblotting was performed as previously described [37]. Immunostaining was performed with anti-SMAD3, anti-phosphorylated SMAD3 and anti-tubulin/actin for loading control.

For immunofluorescence Huh7, HepG2, and 3T3 cells grown in cover slips were fixed in 4% formaldehyde for 20 min and then washed and stained with primary antibodies against E-Cadherin and N-Cadherin (Novus Biological). Alexa Fluor 488 and Alexa 555 (Life Biotechnologies) were used as secondary antibodies, and TO-PRO-3-iodide (Life Biotechnologies) for counterstaining. An Axiovert LSM 510 confocal microscope (Zeiss, Jena, Germany) was used for image collection. Images were analyzed using LSM image browser software (Zeiss).

### Bioinformatics analysis

Raw expression bead array data was exported from Genome Studio (version 2010.3, Illumina) into BRB-ArrayTools software (version 4.3.1, developed by Dr. Richard Simon and the BRB-ArrayTools Development Team), as previously described [37]. Data was normalized and annotated using the R/Bioconductor package “lumi” [38]. Class comparison between groups of bead arrays was done computing a t-test separately for each gene using the normalized log-transformed beta values. Only those probes with FDR < 0.05 and a fold-change of two were considered differentially expressed. WebGestalt (WEB-based GEne SeT AnaLysis Toolkit) web application was used for gene set enrichment analyses, including Gene Ontology, and pathways [39].

For methylome analyses we used a combination of R/Bioconductor packages (following recent guidelines for HM450 data mining) [40, 41]. “WateRmelon” package was used to load the raw data directly from idat files into a MethyLumiSet object [38, 42]. Data quality was inspected using boxplots for the distribution of methylated and unmethylated signals, and inter-sample relationship using multidimensional scaling plots and unsupervised clustering. Probes were filtered for low bead count, low quality (detection P value > 0.05), and recently described cross-reactive probes [43]. Then, we performed color bias adjustment, followed by inter-sample quantile normalization, and probe bias correction with intra-array beta-mixture quantile normalization, as described [44]. Methylation beta values were logarithmically transformed to M values, better suited for parametric statistical analyses [45]. M values were used to determine batch effects using principal component analysis, and corrected with the surrogate variable analysis package (“sva”) [46]. To obtain a common differential methylation between control and TGF-β-treated cells we used the “limma” package with TGF-β exposure as the main variable, using the cell line (Huh7 and HepG2) as a co-factor in the linear model [47]. Differentially methylated positions (DMPs) were defined as those sites with a methylation difference (delta-beta) of 10% in any direction with an FDR-adjusted p value below 0.05. To study the genomic context of DMPs we used HM450 annotations, with hg19 as the human reference genome. Other genomic features were obtained from a recent publication using HM450 bead arrays [23]. Finally, we used the “methyAnalysis” package to identify differentially methylated regions (DMRs) after transforming our dataset to a list of genomic regions followed by methylation data smoothing, as described (Pan Du and Richard Bourgon [2013]. methyAnalysis: DNA methylation data analysis and visualization. R package version 1.2.0). We defined DMR as a region with at least two differentially methylated probes, and a minimum gap of 100 bp.

To study differential methylation between CD133 negative and positive cells we performed a class comparison blocking by cell line status (Huh7 or HepG2). The analysis performed is an analysis of variance for a randomized block design. Two linear models are fit to the methylation data for each gene. The full model includes class variable and the block variable, and the reduced model includes only the block variable. Likelihood ratio test statistics are used to investigate the significance of the difference between the classes. In this comparison we set a p value threshold <0.001 and 5% difference in methylation. For all other comparisons, only those probes with FDR-corrected p values <0.05 were considered significant.

### Statistical analysis

BRBArrayTools and R/Bioconductor packages were used for bead array analyses, as described above. For other comparisons, means and differences of the means with 95% confidence intervals were obtained using GraphPad Prism (GraphPad Software Inc.). Two-tailed student t test was used for unpaired analysis comparing average expression between classes. P values < 0.05 were considered statistically significant.

## Electronic supplementary material

Additional file 1: Figure S1: **A**. Percentage of positive cells for candidate liver cancer stem cell markers, in two unrelated liver cancer cell lines, Huh7 and HepG2. **B**. cell lines were sorted using MACS (as described in Materials and Methods) and RNA was extracted to study the expression of stemness transcription factors (NANOG, POU5F1/Oct4, and SOX2) by qRT-PCR. Intermediate levels of CD133 enrichment were also included, so that increasing expression of CD133 is shown from left to right within each panel. A representative experiment of at least three independent MACS assays per cell line is shown. **C**. sphere formation assay comparing CD133- and CD133+ cells in Huh7 cells. After MACS purification, cells were plated in non-attachment plates, and their growth as spheres was quantified after 6 days. Only structures grown in suspension, with refractory well-defined limits, were counted as spheres. Mean and SD from 3 technical replicates is shown on the left panel. One representative image of each condition is shown on the right panel. (TIFF 580 KB)

Additional file 2: Table S1: Differentially methylated sites in CD133- vs. CD133+ cells, based on Infinium HM450 data. (XLS 338 KB)

Additional file 3: Table S2: Gene set enrichment analyses using BRBArray Tools, and comparing the methylomes of CD133- and CD133+ cells in two cell lines, Huh7 and HepG2. (XLS 119 KB)

Additional file 4: Figure S2: **A**. FACS analysis of TGFBRII expression in Huh7 and HepG2 cells in basal conditions. Percentage of positive cells relative to background secondary antibody is shown in each chart. **B**. western blot for SMAD proteins was performed for the two cell lines, in control conditions, or after stimulation with TGF-β during 4 days. **C**. representative phase contrast images of Huh7 and HepG2 cells left untreated or exposed to IL-6 or TGF-β during 4 days. **D**. viability was assessed by trypan blue exclusion in cells treated or not with IL-6 or TGF-β during the indicated time points. Percentages of trypan positive cells are represented on the bar plots. **E**. Representative phase contrast images of Huh7 and HepG2 cells treated from 1-3 days with the indicated conditions: mock, DMSO, TGF-β receptor I inhibitor (SB-431542), TGF-β alone or in combination with SB-431542 inhibitor. All conditions were performed in triplicate culture wells. **F**. Control and TGF-β -treated cells were fixed and stained for expression of E-Cadherin (FITC) and N-Cadherin (Cy3). E-Cadherin is lost upon treatment in both cell lines and time points (4 and 8 days). N-Cadherin staining was low to absent in all conditions, despite a clear signal in control 3T3 cells (right panel). (TIFF 3 MB)

Additional file 5: Figure S3: **A**. BrdU uptake was used to estimate the proliferation index of both cell lines in different culture conditions, and after two time points. FACS analysis was performed in combination with propidium iodide staining to separate the cells by cell cycle stage. **B**. mRNA expression of CD133 in the same conditions described for Figure 4a. **C**. Non-attachment growth assay was performed after 4 days post-release from a 4 day treatment with TGF-β. Sphere formation was assessed 6 days after culture with hepatosphere medium. (*) indicates P value < 0.05 relative to non-treated. (TIFF 566 KB)

Additional file 6: Table S3: List of differentially methylated sites in response to TGF-β and in two cell lines, Huh7 and HepG2 (TGF-β signature). (XLS 395 KB)

Additional file 7: Table S4: Genes differentially expressed (including gene ontology and pathway analysis) in response to TGF-β. (XLS 258 KB)

Additional file 8: Figure S4: **A**. Correlation between methylation and expression at the genomic regional level in Huh7 cells. Panels show the correlation of delta_Beta (methylation) in the x axis and fold-change (expression) in the y axis. Upper panels correspond to all RefSeq genes without any filter, or separately for CpG-island (CGI) or non-CGI related sites. Lower panels show the same analysis after filtering for differentially methylated and differentially expressed genes. Examples of specific genomic regions (i.e. TSS200, TSS1500, or Gene Body) are listed below the lower panels. The same analysis in HepG2 cells is shown in (**B**). **C**. A selection of significant genes was validated by qRT-PCR in both cell lines. (*) indicates P value < 0.05 relative to non-treated. (TIFF 1 MB)

Additional file 9: Table S5: List of antibodies used for characterization of liver cancer stem cells.**Table S6.** List of pyrosequencing assays. **Table S7.** List of primers used for qRT-PCR. (DOC 73 KB)

## Abbreviations

BMP: Bone morphogenetic protein
BrdU: Bromodeoxyuridine
CGI: CpG island
TIC: Tumor-initiating cell
EMT: Epithelial-mesenchymal transition
FACS: Fluorescence-activated cell sorting
HCC: Hepatocellular carcinoma
IL6: Interleukin 6
TGF-β: Transforming growth factor beta.
