# Supplementary figures and images for: Dynamic imbalance between cancer cell subpopulations induced by Transforming Growth Factor Beta (TGF-β) is associated with a DNA methylome switch
Source: BMC Genomics. 2014 Jun 5;15(1):435. doi: 10.1186/1471-2164-15-435 (PMC4070873; doi:10.1186/1471-2164-15-435)

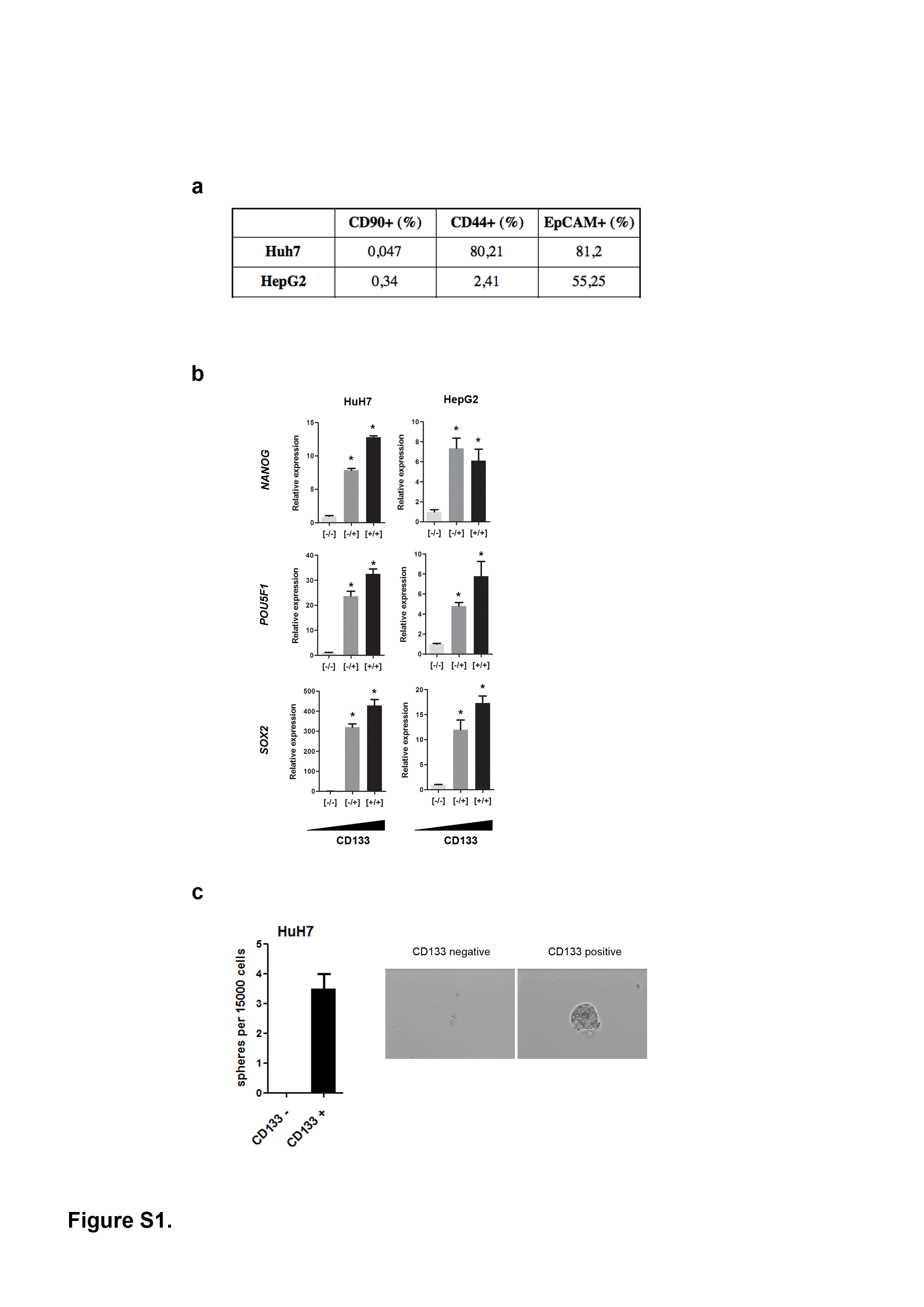

Supplement: Supplementary file 1 — Additional file 1: Figure S1: A. Percentage of positive cells for candidate liver cancer stem cell markers, in two unrelated liver cancer cell lines, Huh7 and HepG2. B. cell lines were sorted using MACS (as described in Materials and Methods) and RNA was extracted to study the expression of stemness transcription factors (NANOG, POU5F1/Oct4, and SOX2) by qRT-PCR. Intermediate levels of CD133 enrichment were also included, so that increasing expression of CD133 is shown from left to right within each panel. A representative experiment of at least three independent MACS assays per cell line is shown. C. sphere formation assay comparing CD133- and CD133+ cells in Huh7 cells. After MACS purification, cells were plated in non-attachment plates, and their growth as spheres was quantified after 6 days. Only structures grown in suspension, with refractory well-defined limits, were counted as spheres. Mean and SD from 3 technical replicates is shown on the left panel. One representative image of each condition is shown on the right panel. (TIFF 580 KB) [file 12864_2013_6137_MOESM1_ESM.tiff]

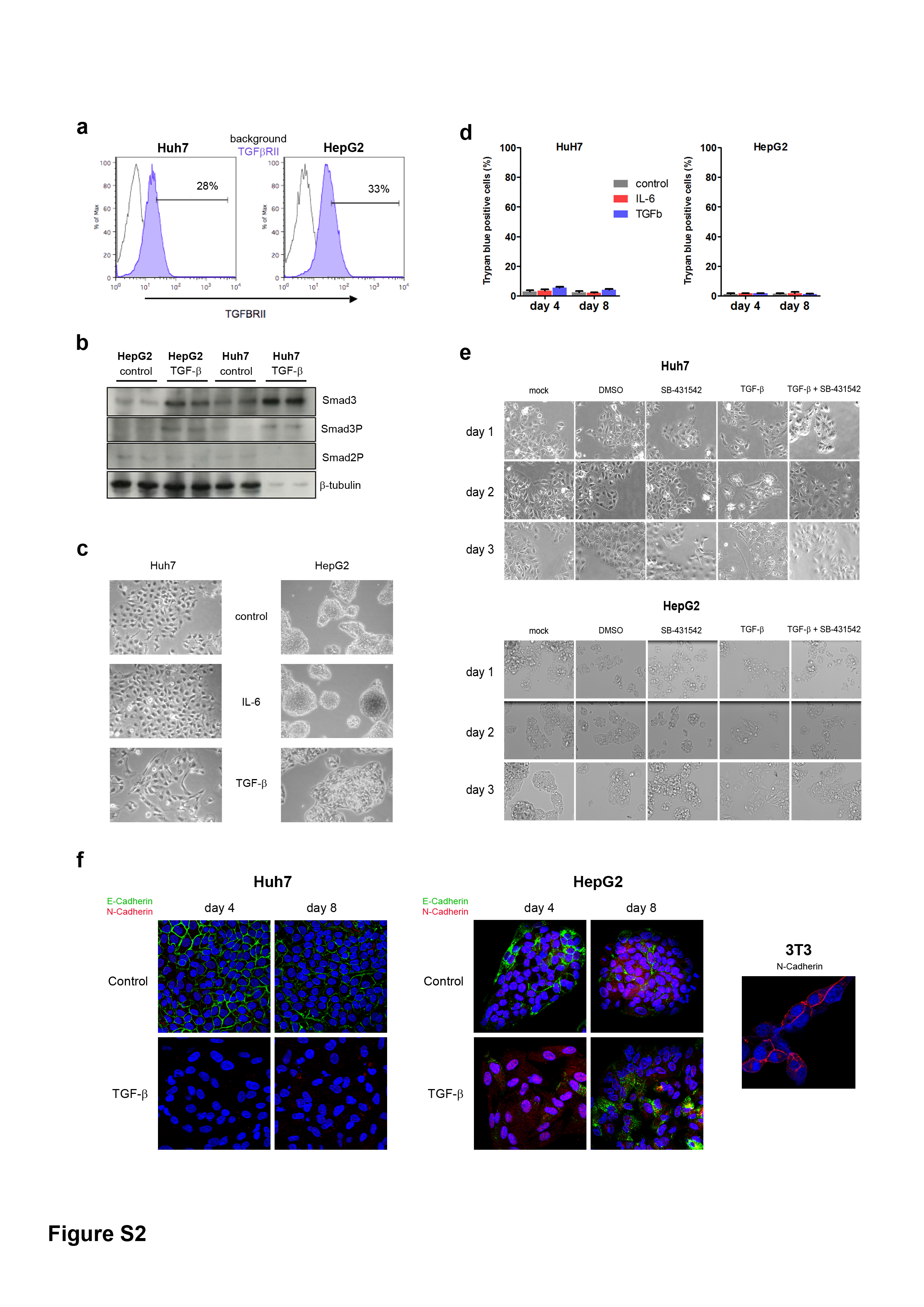

Supplement: Supplementary file 4 — Additional file 4: Figure S2: A. FACS analysis of TGFBRII expression in Huh7 and HepG2 cells in basal conditions. Percentage of positive cells relative to background secondary antibody is shown in each chart. B. western blot for SMAD proteins was performed for the two cell lines, in control conditions, or after stimulation with TGF-β during 4 days. C. representative phase contrast images of Huh7 and HepG2 cells left untreated or exposed to IL-6 or TGF-β during 4 days. D. viability was assessed by trypan blue exclusion in cells treated or not with IL-6 or TGF-β during the indicated time points. Percentages of trypan positive cells are represented on the bar plots. E. Representative phase contrast images of Huh7 and HepG2 cells treated from 1-3 days with the indicated conditions: mock, DMSO, TGF-β receptor I inhibitor (SB-431542), TGF-β alone or in combination with SB-431542 inhibitor. All conditions were performed in triplicate culture wells. F. Control and TGF-β -treated cells were fixed and stained for expression of E-Cadherin (FITC) and N-Cadherin (Cy3). E-Cadherin is lost upon treatment in both cell lines and time points (4 and 8 days). N-Cadherin staining was low to absent in all conditions, despite a clear signal in control 3T3 cells (right panel). (TIFF 3 MB) [file 12864_2013_6137_MOESM4_ESM.tiff]

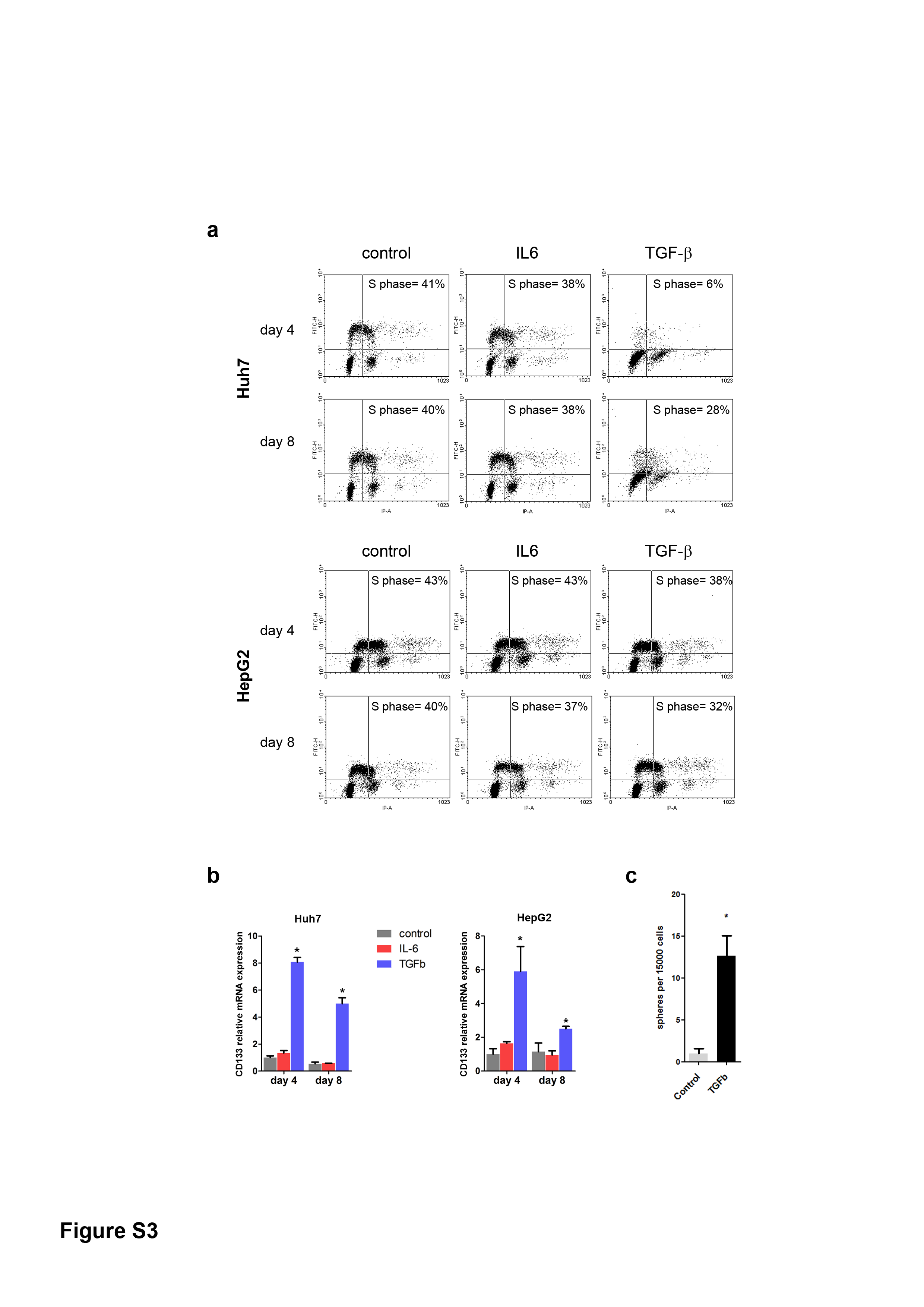

Supplement: Supplementary file 5 — Additional file 5: Figure S3: A. BrdU uptake was used to estimate the proliferation index of both cell lines in different culture conditions, and after two time points. FACS analysis was performed in combination with propidium iodide staining to separate the cells by cell cycle stage. B. mRNA expression of CD133 in the same conditions described for Figure 4a. C. Non-attachment growth assay was performed after 4 days post-release from a 4 day treatment with TGF-β. Sphere formation was assessed 6 days after culture with hepatosphere medium. (*) indicates P value < 0.05 relative to non-treated. (TIFF 566 KB) [file 12864_2013_6137_MOESM5_ESM.tiff]

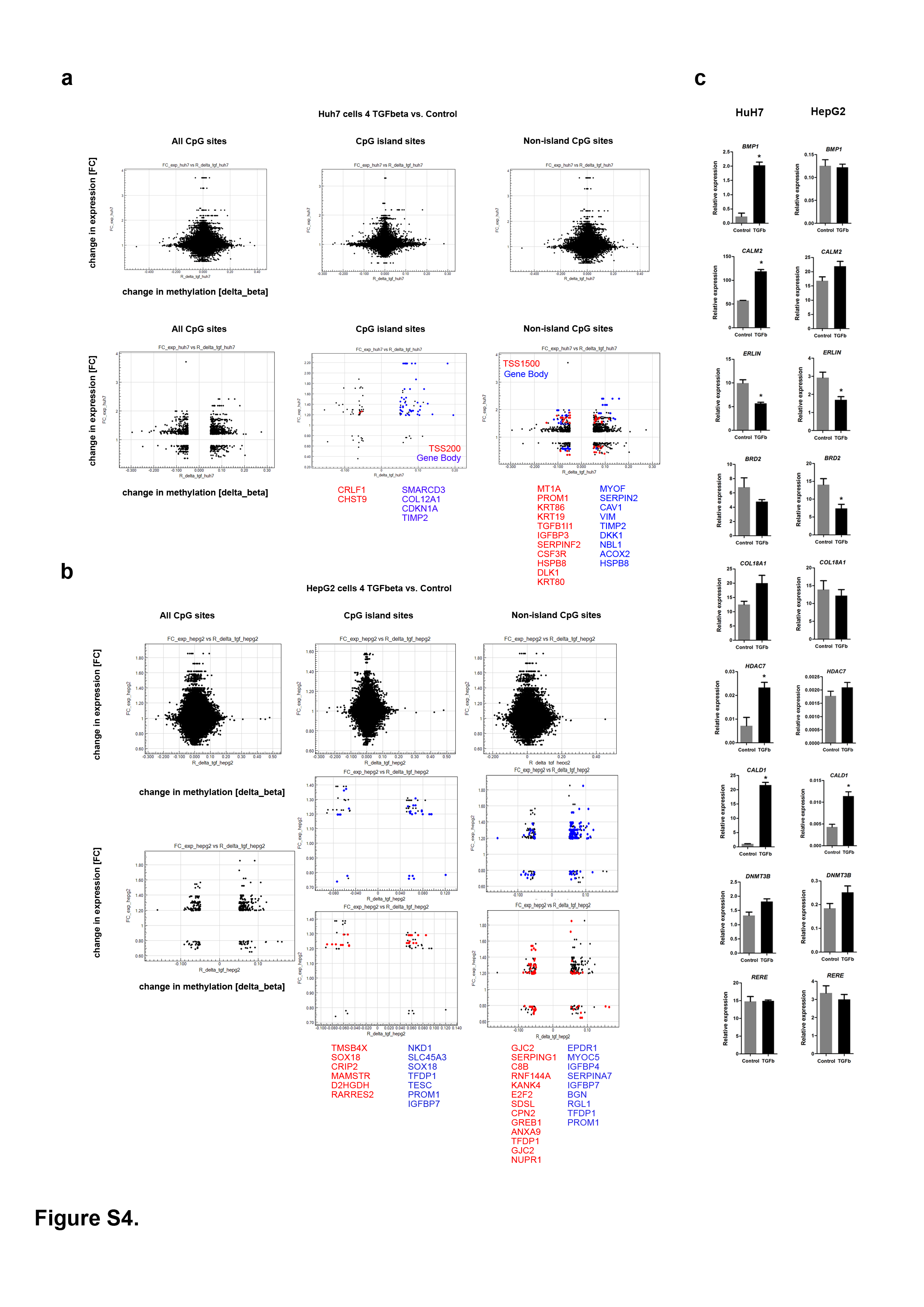

Supplement: Supplementary file 8 — Additional file 8: Figure S4: A. Correlation between methylation and expression at the genomic regional level in Huh7 cells. Panels show the correlation of delta_Beta (methylation) in the x axis and fold-change (expression) in the y axis. Upper panels correspond to all RefSeq genes without any filter, or separately for CpG-island (CGI) or non-CGI related sites. Lower panels show the same analysis after filtering for differentially methylated and differentially expressed genes. Examples of specific genomic regions (i.e. TSS200, TSS1500, or Gene Body) are listed below the lower panels. The same analysis in HepG2 cells is shown in (B). C. A selection of significant genes was validated by qRT-PCR in both cell lines. (*) indicates P value < 0.05 relative to non-treated. (TIFF 1 MB) [file 12864_2013_6137_MOESM8_ESM.tiff]
