# Supplementary material for: Dynamic imbalance between cancer cell subpopulations induced by Transforming Growth Factor Beta (TGF-β) is associated with a DNA methylome switch
Source: BMC Genomics. 2014 Jun 5;15(1):435. doi: 10.1186/1471-2164-15-435 (PMC4070873; doi:10.1186/1471-2164-15-435)
Supplement: Supplementary file 9 — Additional file 9: Table S5: List of antibodies used for characterization of liver cancer stem cells.Table S6. List of pyrosequencing assays. Table S7. List of primers used for qRT-PCR. (DOC 73 KB) [file 12864_2013_6137_MOESM9_ESM.doc]

*Table S5* : list of antibodies used for fluorescent activated cell sorting (FACS)

| Primary antibodies | Fluorochrome | Isotype and origin | Company | Working dilution |
| --- | --- | --- | --- | --- |
| Anti-human CD44 | Pe conjugated | G44-26 Mouse IgG2b | BD Pharmingen | 1:10 |
| Anti-human CD133 | Non conjugated | AC133 mouse IgG1 | Miltenyi Biotec | 1:10 |
| Anti-human EpCAM | PerCP conjugated | clone 1B7 mouse IgG1 | Ebiosciences | 1:50 |
| Anti-human CD90 | FITC conjugated | 5E10 mouse IgG1 | Stem cell technologies | 1:10 |
| Anti-human TGFBRII | Pe-conjugated | Goat | RD system | 1/10 |
|  |  |  |  |  |
| Secondary antibodies |  |  |  |  |
| Anti-mouse-FITC | - | - | Sigma | 1:100 |
| Anti-mouse Cy3 | - | - | Sigma | 1:200 |
| Anti-mouse-Alexa 750 | - | - | Invitrogen | 1:100 |
|  |  |  |  |  |

*Table S6. list of primers design for pyrosequencing assays*

| ***Genes*** | ***Primers for PCR*** | ***UCSC localization*** | ***Annealing temperature*** | ***Sequencing primers*** | ***Sequence analyzed*** | ***strand*** | ***Infinium450K probe***  ***correspondance*** |
| --- | --- | --- | --- | --- | --- | --- | --- |
| *•DNMT1* | For GTATTTGGGGATTAAAAGAG  Rev Btn-TTAATACATCCCCTCCTC | Chr19:10,305,553-10,305,597 | 49 | GAAGTGTTATTTTGTT | CGCGTTAATTGTCGTTGCGCG | + |  |
| *DNMt3a (assay1)* | For TTAGTATTGGGGTTGGGGATAGTAG  Rev Btn-CACTCCCTTCAAAACAACTCATCTC | chr2:25,565,470-25,565,648 | 60 | TTATAGGGTTAAGGT | YGGAGYGTTAGGTTTTTTTTGGT | + |  |
| *DNMT3a (assay2)* | For TGAGTTTGGGGAGAGGAG  Rev btn-CCCCAACCTACCTACTACAAA | chr2:25,481,174-25,481,364 | 59 | TTTAGAATTTGTAAG | YGAATTGTGTTTTATT | + | cg04058399 |
| *DNMT3b (assay1)* | For AGGGTTGTTAGGGGAGGGGA  Rev btn-CCTCACCTCCTCTACCCCTT | chr20:31,350,050-31,351,0363 | 61 | ATTTTGTTTGGGGTGGAA | YGGGGATAGYGGGTGGGAYGYGGGG | + |  |
| *DNMT3b (assay2)* | For GGTAGGTGTAGGGTTTGGA  Rev Btn-GGTAGGTGTAGGGTTTGGA | chr20:31,366,350-31,366,545 | 57 | TATTAGATTGAGTTTA  GTGTAGTTTGGAGTT | YGGTGTTTTTTGGTGGGYGATGTT  YGTAATATAAGGTGTGG | + | cg24403338  cg00300969 |
| *TRRAP* | For GTGGTTTGGAATTGTTTTAGG  Rev Btn-ATCTATACCAACTTCTCCCTCC | chr7:98,520,412-98,520,578 | 57 | TATTTTTGTAGAAGA | YGTGTGTTTATGTAGT | + | cg21421984 |
| *TET2* | For AGGGTAGGAATGGGTTAG  Rev Btn-CTATCTTCCCTTCTCTCTTAAC | chr4:106,114,429-106,114,753 | 53 | GAAGTAGGAAAAAGG | YGATTTTGTATAAA | + | cg22794775 |
| *CD133* | For GTGGGGATTTGTTTTAGTTA  Rev Btn-CCACTAACATTAATACTACTACACAC | chr4:16,085,637-16,086,016 |  | TATTTTGATTGGAT | YGGTTGAGTTGTTTYGTTAYGTAGTTTTGGG | + | cg04203238 |

*Table S7: list of primers used for quantitative PCR experiments*

| *HPRT1* | *for* 5’-CATTGTAGCCCTCTGTGTGC-3’  *rev* 5’-CACTATTTCTATTCAGTGCTTTGATGT-3’ | *SOX2* | *for* 5’-AAGACGCTCATGAAGAAGGATAA-3’  *rev* 5’-ACTGTCCATGCGCTGGTT-3’ |
| --- | --- | --- | --- |
| *GAPDH* | *for* 5’-AACGGGAAGCTTGTCATCAA-3’  *rev* 5’-TGGACTCCACGACGTACTCA-3’ | *BMP1* | *for* 5’-Caaggcccacttcttctcag-3’  *rev* 5’-cataactgccgaacgtgttg-3’ |
| *SFSR4* | *for* 5’-GGCTACGGGAAGATCCTGGA-3’  *rev* 5’-TGCATCACGCAGATCATCAA-3’ | *ERLIN1* | *for* 5’-gattgaggagggccatctg-3’  *rev* 5’-ggtccactggggctagttagt-3’ |
| *TBP1* | *for* 5’-TATAATCCCAAGCGGTTTGC-3’  *rev* 5’-CACAGCTCCCCACCATATTC-3’ | *HDAC7* | *for* 5’-ggtgtcctagacgcacagaaat-3’  *rev* 5’-catgaccgagtcatagatcagc-3’ |
| *CD133* | *for* 5’-TCCACAGAAATTTACCTACATTGG-3’  *rev* 5’-CAGCAGAGAGCAGATGACCA-3’ | *RERE* | *for* 5’-tgaagaagtcggccaagaag-3’  *rev* 5’-cgctggcgtttgttactctt-3’ |
| *DNMT3a* | *for* 5’-CCTGAAGCCTCAAGAGCAGT-3’  *rev* 5’-TGGTCTCCTTCTGTTCTTTGC-3’ | *ZEB1* | *for* 5’-gctgggaggatgacagaaag-3’  *rev* 5’-tgcatctgactcgcattcat-3’ |
| *DNMT3b* | *for* 5’-CAAATGGCTTCAGATGTTGC-3’  *rev* 5’-TCCTGCCACAAGACAAACAG-3’ | *COL18A1* | *for* 5’-aggaaggactgggcagaaa-3’  *rev* 5’-ctcccttgctccccttatgt-3’ |
| *DNMT1* | *for* 5’-GATGTGGCGTCTGTGAGGT-3’  *rev* 5’-CCTTGCAGGCTTTACATTTCC-3’ | *CALD1* | *for* 5’-cgtcgcagagaacttagaagg-3’  *rev* 5’-attcctctggtaggcgattct-3’ |
| *TET1* | *for* 5’-GCTATACACAGAGCTCACAG-3’  *rev* 5’-GCCAAAAGAGAATGAAGCTCC-3’ | *CALM2* | *for* 5’-atggctgaccaactgactga-3’  *rev* 5’-cagttcccaattcctttgttg-3’ |
| *TET2* | *for* 5’-CTTTCCTCCCTGGAGAACAGCTC-3’  *rev* 5’-TGCTGGGACTGCTGCATGACT-3’ | *BRD2* | *for* 5’-CCCTAAGAACAGCCACAGAA-3’  *rev* 5’-GGTATCTCAGGTGGAGGAGTAT-3’ |
| *TGFb* | *for* 5’-GCACGTGGAGCTGTACCA-3’  *rev* 5’-AAGATAACCACTCTGGCGAGTC-3’ | *STAT3* | *for* 5’-AACTTCAGACCCGTCAACAAA-3’  *rev* 5’-GGGTCCCCTTTGTAGGAAAC-3’ |
| *SNAIL* | *for* 5’-ATCCGAAGCCACACACTG-3’  *rev* 5’-CACTGGTACTTCTTGACATCTG-3’ | *JAK2* | *for* 5’-GGTGAAAGTCCCATATTCTGGT-3’  *rev* 5’-AGGCCACAGAAAACTTGCTC-3’ |
| *P21* | *for* 5’-GACACCACTGGAGGGTGACT-3’  *rev* 5’-CCACATGGTCTTCCTCTGCT-3’ | *NANOG* | *for* 5’-CAGCTGTGTGTACTCAATGATAGATTT-3’  *rev* 5’-TCTGGAACCAGGTCTTCACC-3’ |
| *CCDN1* | *for* 5’-GAAGATCGTCGCCACCTG-3’  *rev* 5’-GACCTCCTCCTCGCACTTCT-3’ | *OCT4* | *for* 5’-GCTTCGGATTTCGCCTTC-3’  *rev*5’-CTTAGCCAGGTCCGAGGAT-3’ |
